# Deep learning reconstruction enhances image quality in contrast-enhanced CT venography for deep vein thrombosis

**DOI:** 10.1007/s10140-025-02366-x

**Published:** 2025-07-18

**Authors:** Yusuke Asari, Koichiro Yasaka, Joji Kurashima, Akira Katayama, Mariko Kurokawa, Osamu Abe

**Affiliations:** Department of Radiology, The University of Tokyo, Tokyo, Japan

**Keywords:** Deep vein thrombosis, Contrast-enhanced CT venography, Deep learning reconstruction, Image quality

## Abstract

**Purpose:**

This study aimed to evaluate and compare the diagnostic performance and image quality of deep learning reconstruction (DLR) with hybrid iterative reconstruction (Hybrid IR) and filtered back projection (FBP) in contrast-enhanced CT venography for deep vein thrombosis (DVT).

**Methods:**

A retrospective analysis was conducted on 51 patients who underwent lower limb CT venography, including 20 with DVT lesions and 31 without DVT lesions. CT images were reconstructed using DLR, Hybrid IR, and FBP. Quantitative image quality metrics, such as contrast-to-noise ratio (CNR) and image noise, were measured. Three radiologists independently assessed DVT lesion detection, depiction of DVT lesions and normal structures, subjective image noise, artifacts, and overall image quality using scoring systems. Diagnostic performance was evaluated using sensitivity and area under the receiver operating characteristic curve (AUC). The paired *t*-test and Wilcoxon signed-rank test compared the results for continuous variables and ordinal scales, respectively, between DLR and Hybrid IR as well as between DLR and FBP.

**Results:**

DLR significantly improved CNR and reduced image noise compared to Hybrid IR and FBP (*p* < 0.001). AUC and sensitivity for DVT detection were not statistically different across reconstruction methods. Two readers reported improved lesion visualization with DLR. DLR was also rated superior in image quality, normal structure depiction, and noise suppression by all readers (*p* < 0.001).

**Conclusions:**

DLR enhances image quality and anatomical clarity in CT venography. These findings support the utility of DLR in improving diagnostic confidence and image interpretability in DVT assessment.

**Graphical Abstract:**

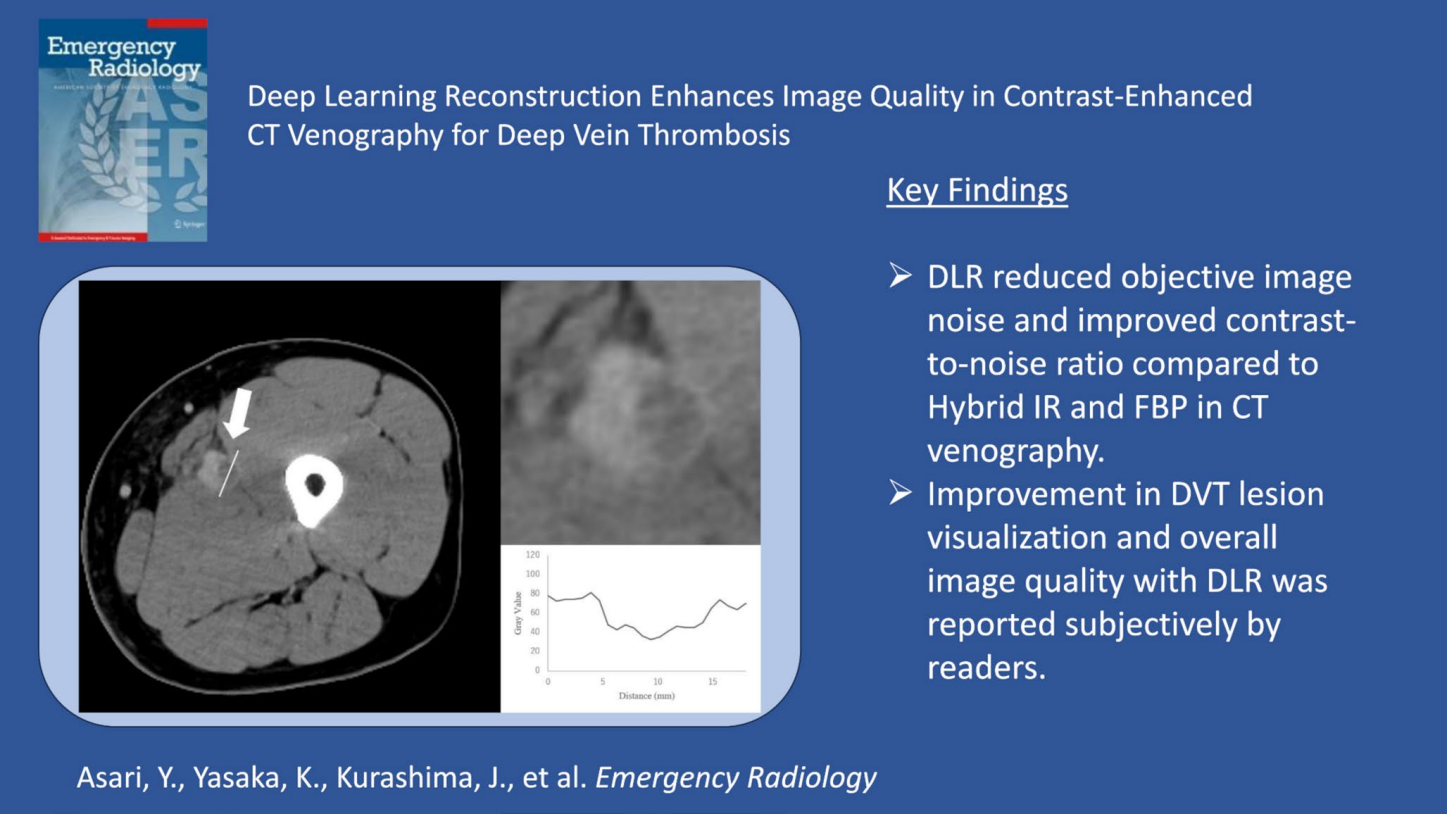

**Supplementary Information:**

The online version contains supplementary material available at 10.1007/s10140-025-02366-x.

## Introduction

Deep vein thrombosis (DVT) is a severe vascular condition that can result in serious complications, including pulmonary embolism. It occurs when blood clots form within the deep venous system, primarily affecting the lower limbs. These clots can obstruct blood flow, causing symptoms such as localized swelling and discomfort [1]. Timely identification and management of DVT are crucial to minimize the risk of complications. Ultrasound remains the primary imaging techniques for detecting DVT due to its accessibility and non-invasive nature. However, its accuracy can be influenced by factors such as operator proficiency and limitations in visualizing specific anatomical regions. Additionally, performing ultrasound in patients with open wounds, bandages, or casts can be challenging. In this context, contrast-enhanced CT serves as a more precise imaging method [2]. It enables simultaneous evaluation of inferior vena cava, pelvis venous embolism, and pulmonary embolism [3]. Nevertheless, CT imaging for DVT presents challenges, including metal artifacts, inadequate contrast enhancement, and low contrast-to-noise ratios in certain areas [2, 4]. Especially, inadequate opacification of distal lower limb vessels due to delayed distal run off sometimes compromise the diagnostic quality of CT venography. Reducing the tube voltage in CT imaging enhances contrast enhancement and contrast-to-noise ratios but also increases image noise [5].

Deep learning has gained traction within radiological imaging in recent years [6, 7]. Its applications span various tasks, including anomaly detection [8], differential diagnosis support [9], and disease staging [10]. Moreover, advancements have demonstrated its effectiveness in improving image quality [11]. One notable application is deep learning reconstruction (DLR), a method that has demonstrated enhanced lesion visibility, reduced image noise, and superior image quality compared to conventional hybrid iterative reconstruction (Hybrid IR) and filtered back projection (FBP) techniques [12–15]. In CT venous imaging, minimizing noise is beneficial for enhancing image quality [16, 17] Therefore, DLR holds potential to improve DVT lesion detection and its depiction, as well as overall image quality.

The objective of this study was to evaluate and compare DVT lesion detection and image quality in contrast-enhanced CT when employing DLR versus Hybrid IR and FBP.

## Materials and methods

### Study design

This retrospective single-center study was approved by the Institutional Review Board of our institution, which waived the requirement for written informed consent due to the retrospective nature of the study.

**Fig. 1:**
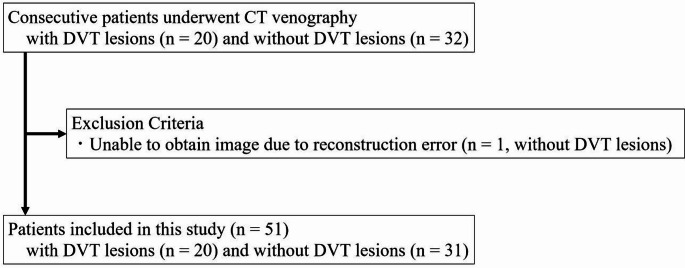
Patient inclusion flowchart. DVT, deep vein thrombosis

We collected image data of patients who underwent contrast-enhanced CT venography of lower limbs. Consecutive patients with DVT lesions between September 2023 and September 2024 (*n* = 32) and those without DVT lesions between September 2023 and February 2024 (*n* = 20) were included (Fig. 1). One patient without DVT was excluded from the study due to reconstruction error.

### CT protocol

CT scans were performed using a multi-detector row CT (Aquilion One; Canon Medical Systems, Otawara, Japan). CT scanning parameters were as follows: scan mode: helical; tube voltage: 100 kVp; tube current: automatic tube current modulation with a standard deviation set at 13.0; helical pitch of 0.813:1. Iodinated contrast media at 600 mgI/kg was intravenously injected using a power injector. The injection rate was 20 mgI/kg/second (injection time: 30 s) and it was consistent across all patients. The CT venography was obtained 210 s after starting the injection. Images were reconstructed using the following algorithms from the raw data: DLR (Advanced intelligent Clear-IQ Engine with body sharp standard, Canon Medical Systems) and Hybrid IR (AIDR 3D enhanced standard with the kernel of FC03, Canon Medical Systems) and FBP with the kernel of FC03. The following image reconstruction parameters were consistent across all image sets: field of view: 350 mm (adjusted to the patient’s body size) and slice thickness/interval: 3/3 mm.

The Advanced Intelligent Clear-IQ Engine utilizes a vendor-trained convolutional neural network (CNN) architecture, specifically designed for noise reduction. The network was trained using a proprietary dataset comprising several clinical high-quality 120 kVp scans of the body in 10 patients. The validation dataset contained three independent image categories: patient images acquired at different dose levels, hepatic and pelvic phantom images obtained at varying dose levels, and images of metal objects and truncation artifacts [18].

### Reference standard

Determination for correct DVT lesions was made through a consensus between one board-certified radiologist with 15 years of imaging experience and one radiology resident with 3 years of imaging experience.

### Quantitative image quality analyses

**Fig. 2:**
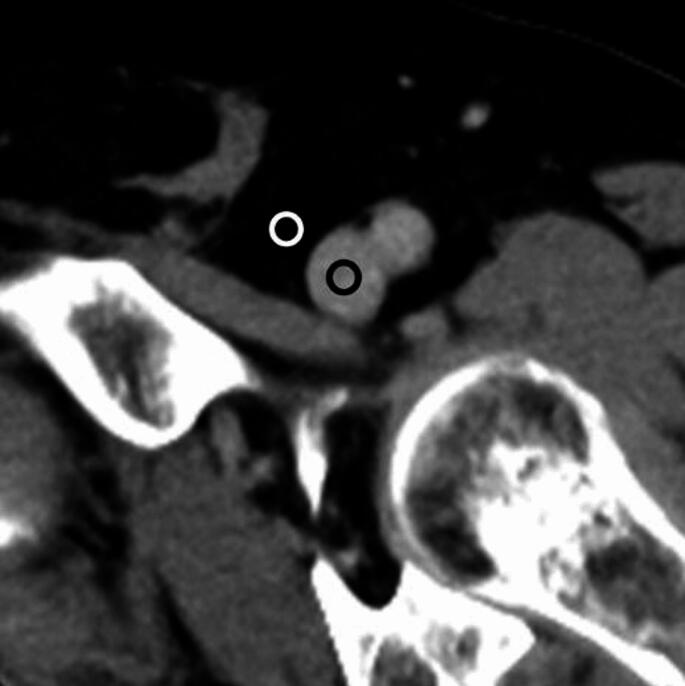
Circular or ovoid ROIs were delineated on the left femoral vein (black circle) and subcutaneous fat (white circle) on the same axial slice. ROI, region of interest

Quantitative image quality analyses were conducted on the contrast-enhanced CT images by a radiology resident with 3 years of imaging experience using ImageJ (https://imagej.net/ij/). Circular or ovoid regions of interest (ROIs) measuring 3–5 mm in diameter were placed on the left femoral vein and subcutaneous fat on the same slice near the level of inguinal ligament (Fig. 2). The apparent lesion was avoided when placing regions of interest on these normal structures. The mean and standard deviation of the CT attenuation for each ROI were calculated and documented. The standard deviation of the CT attenuation for normal structures was considered quantitative image noise. The following metric were then computed:

CNR = (MA_VEIN_ − MA_FAT_)/√((SD_VEIN_² + SD_FAT_²)/2).

CNR, MA_FAT_, MA_VEIN_, SD_FAT_, and SD_VEIN_ refer to the contrast-to-noise ratio, mean attenuation for subcutaneous fat and femoral vein, and standard deviation of the attenuation for subcutaneous fat and femoral vein, respectively. For the cases with DVT lesion, additional CNR between the thrombus and adjacent contrast-opacified vein lumen was calculated. The size of the ROI was selected so that it would not be larger than the size of the thrombus or vein lumen. The location and size of the ROIs were kept consistent between DLR, Hybrid IR, and FBP.

### Lesion detection and qualitative image analyses

A board-certified radiologist with 15 years of imaging experience randomized all image sets. An additional radiologist (Readers 1, with 8 years of imaging experience) and two radiology residents (Reader 2 and 3, with 4 and 2 years of imaging experience, respectively) independently analyzed the contrast-enhanced CT images using ImageJ. They were blinded to the patient details and reconstruction methods.

The readers were asked to determine whether each patient had any DVT lesions and recorded confidence scores on a four-point scale (4, definitely present; 3, probably present; 2, uncertain for the presence or absence; and 1, no lesion). The test was conducted in three sessions, ensuring that DLR, Hybrid IR, and FBP images for the same patient did not appear in the same session. A 2-week interval was maintained between sessions to prevent recall bias.

The three same readers assessed the contrast-enhanced CT images at a minimum of 2 weeks after the lesion detection test. The images were assessed using the following criteria:

- DVT lesion depiction (all lesions were annotated and assessed) (4, clear depiction; 3, slightly blurred; 2, moderately blurred; and 1, unrecognizable).
- Structure depiction (veins and muscles) (4, clear depiction; 3, slightly blurred; 2, moderately blurred; and 1, unrecognizable).
- Subjective image noise (4, less noise; 3, standard noise; 2, more than standard noise; and 1, severe noise).
- Presence of artifacts (3, almost no artifacts; 2, mild artifacts; 1, severe artifacts affecting imaging diagnosis).
- Overall image quality (5, excellent; 4, better than standard; 3, standard; 2, worse than standard; and 1, poor).

### Statistical analysis

Statistical analyses were conducted using R (version 4.3.1; https://www.r-project.org/). The *t*-test and Fisher’s exact test were employed to compare demographic characteristics between the DVT lesion and non-DVT lesion groups. The paired *t*-test and Wilcoxon signed-rank test were utilized to compare the results for continuous variables and ordinal scales, respectively, between DLR and Hybrid IR, as well as between DLR and FBP. Diagnostic performance for lesion detection was calculated using the area under the receiver operating characteristic curve (AUC) and was subsequently compared with the DeLong test. To assess the sensitivity of the detection test, diagnostic confidence scores of ≥ 2 were considered indicative of lesion presence. These scores were evaluated statistically using McNemar’s test. Subgroup analyses of AUC and sensitivity were also performed based on lesion length (> 5 cm vs. <5 cm), lesion diameter (> 7 mm vs. <7 mm), location (femoral vs. peripheral), and occlusion grade (complete vs. partial) [19]. The interobserver agreement for lesion detection between the three readers was evaluated with the Fleiss’ kappa analysis. Kappa values of 0.00–0.20, 0.21–0.40, 0.41–0.60, 0.61–0.80, and 0.81–1.00 indicated slight, fair, moderate, substantial, and excellent agreement, respectively [20]. *P*-values < 0.025 were used to denote statistical significance after Bonferroni correction.

## Results

### Patient characteristics

Table 1 presents background details for the study participants. A total of 51 patients (mean age: 67.0 ± 15.0 years; 26 males, 25 females) were subsequently analyzed. Of these, 20 presented with 30 DVT lesions, while 31 did not. No statistically significant differences were observed in age or sex between the DVT lesion group and the non-lesion group. The distribution of lesions was as follows: 2 patients had 4 lesions, 4 had 2 lesions, and 14 had 1 lesion.

**Table 1:** Demographic characteristics of patients in the DVT lesion and non-lesion groups

	DVT lesion group (*n* = 20)	Non-lesion group (*n* = 31)	*P* value
Age (years: mean ± standard deviation)	70.5 ± 12.2	64.7 ± 16.3	0.180
Sex (male, female)	(10,10)	(16,15)	> 0.99

Age; *t*-test. Sex; Fisher’s exact test

DVT, deep vein thrombosis

### Quantitative image quality analysis

Table 2 presents the quantitative evaluations. DLR significantly reduced image noise across all anatomical structures compared to Hybrid IR and FBP (*p* < 0.001 for all comparisons). The CNR between femoral vein and subcutaneous fat was notably better with DLR (21.4) than with Hybrid IR (17.5) and FBP (10.0), with statistical significance (*p* < 0.001). For the cases with DVT lesion, the CNR between the thrombus and adjacent contrast-opacified vein lumen was also higher with DLR (4.0) than with Hybrid IR (3.3) and FBP (2.4), with significant difference (*p* < 0.001).

**Table 2:** Results of quantitative image analysis

	DLR	Hybrid IR	FBP	*P* value (vs. Hybrid IR, vs. FBP)
Image noise (fat)	10.0 ± 3.5	11.6 ± 3.6	19.2 ± 4.3	< 0.001 *, < 0.001 *
Image noise (vein)	8.0 ± 1.9	10.6 ± 2.8	19.4 ± 4.1	< 0.001 *, < 0.001 *
CNR	21.4 ± 5.9	17.5 ± 4.5	10.0 ± 2.0	< 0.001 *, < 0.001 *

CNR, contrast-to-noise ratio; DLR, deep learning reconstruction; FBP, filtered back projection; IR, iterative reconstruction

*, statistically significant difference

### Lesion detection and qualitative image quality assessment

**Fig. 3:**
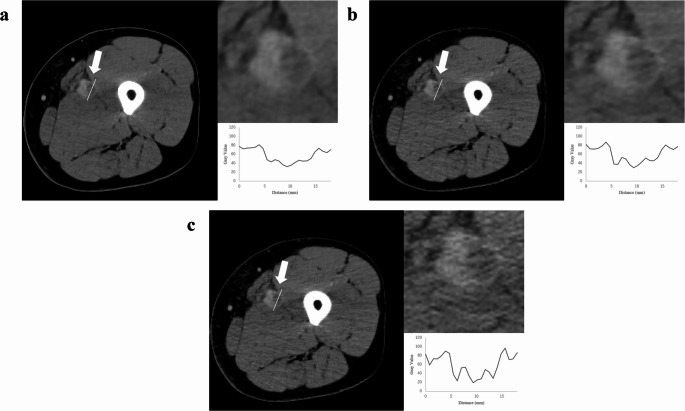
CT venography images reconstructed using deep learning reconstruction (**a**), hybrid iterative reconstruction (**b**), and filtered back projection (**c**) techniques. The images depict adeep vein thrombosis lesion (left, arrow) observed in a 47-year-old male patient. Zoomed-in insets (top right) and signal profile plots (bottom right) across lesions (left, bar)

**Fig. 4:**
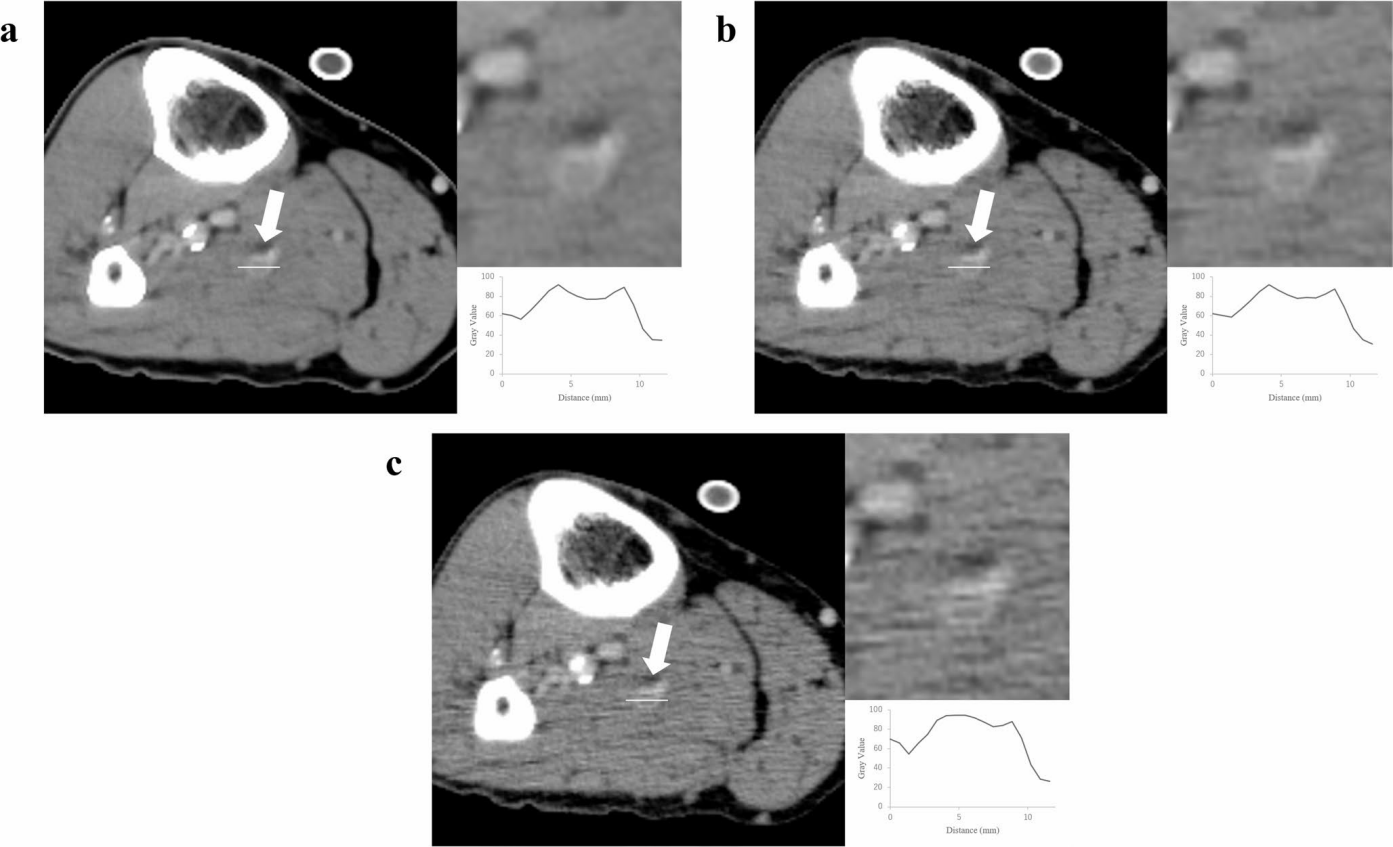
CT venography images reconstructed using deep learning reconstruction (**a**), hybrid iterative reconstruction (**b**), and filtered back projection (**c**) techniques. The images depict a deep vein thrombosis lesion (left, arrow) observed in a 72-year-old male patient. Zoomed-in insets (top right) and signal profile plots (bottom right) across lesions (left, bar)

The outcomes of lesion detection are presented in Table 3. There were no statistically significant differences in the AUC and sensitivity between DLR and Hybrid IR or FBP. Tables 4 and 5 present the results of the subgroup analyses of AUC and sensitivity based on lesion length, diameter, location, and occlusion grade. No statistically significant differences were observed. The result of inter-reader agreement was moderate (0.417). As shown in Table 6, qualitative evaluations indicated that readers 1 and 2 rated visualization of DVT as significantly improved in DLR compared to Hybrid IR and FBP (*p* ≤ 0.020). For depiction of veins and muscles, all readers rated DLR superior, (*p* < 0.001). Subjective image noise was also reduced in DLR compared to the other two methods (*p* < 0.001). Regarding artifacts, the readers identified fewer artifacts (*p* ≤ 0.005), except readers 2 did not find a significant difference. Overall, DLR was rated as having better image quality by all readers (*p* < 0.001). The example images of DLR, Hybrid IR, and FBP are presented in Figs. 3 and 4.

**Table 3:** Results of lesion detection analysis

	Reader	DLR	Hybrid IR	FBP	*P* value (vs. Hybrid IR, vs. FBP)
AUC	1	0.798	0.773	0.802	0.627, 0.956
	2	0.724	0.772	0.740	0.383, 0.675
	3	0.714	0.710	0.684	0.923, 0.494
Sensitivity	1	0.700	0.700	0.700	> 0.99, > 0.99
	2	0.600	0.650	0.650	> 0.99, > 0.99
	3	0.550	0.500	0.500	> 0.99, > 0.99

Comparisons were conducted using the DeLong test for AUC and McNemar’s test for sensitivity

AUC, area under the receiver operating characteristic curve; DLR deep learning reconstruction; FBP, filtered back projection; IR, iterative reconstruction

**Table 4:** Results of lesion detection subgroup analysis (AUC)

	Reader	DLR	Hybrid IR	FBP	*P* value (vs. Hybrid IR, vs. FBP)
Length					
>5 cm	1	0.917	0.844	0.955	0.454, 0.207
	2	0.955	0.966	0.955	0.317, > 0.99
	3	0.932	0.966	0.946	0.076, 0.552
<5 cm	1	0.614	0.629	0.595	0.771, 0.790
	2	0.498	0.551	0.518	0.441, 0.671
	3	0.499	0.473	0.452	0.634, 0.391
Diameter					
> 7 mm	1	0.814	0.751	0.795	0.331, 0.711
	2	0.859	0.874	0.834	0.282, 0.267
	3	0.788	0.822	0.801	0.150, 0.602
< 7 mm	1	0.624	0.656	0.651	0.563, 0.765
	2	0.450	0.511	0.505	0.503, 0.338
	3	0.516	0.470	0.451	0.501, 0.344
Location					
Femoral	1	0.926	0.753	0.926	0.259, > 0.99
	2	0.926	0.936	0.926	0.317, > 0.99
	3	0.904	0.936	0.936	0.077, 0.176
Peripheral	1	0.688	0.718	0.691	0.515, 0.956
	2	0.605	0.654	0.623	0.415, 0.669
	3	0.601	0.586	0.557	0.753, 0.358
Occlusion					
Complete	1	0.760	0.688	0.791	0.402, 0.343
	2	0.785	0.799	0.779	0.321, 0.774
	3	0.782	0.814	0.791	0.141, 0.732
Partial	1	0.704	0.724	0.686	0.679, 0.817
	2	0.588	0.640	0.613	0.472, 0.600
	3	0.576	0.547	0.530	0.604, 0.409

Comparisons were conducted using the DeLong test

AUC, area under the receiver operating characteristic curve; DLR deep learning reconstruction; FBP, filtered back projection; IR, iterative reconstruction

**Table 5:** Results of lesion detection subgroup analysis (Sensitivity)

	Reader	DLR	Hybrid IR	FBP	*P* value (vs. Hybrid IR, vs. FBP)
Length					
>5 cm	1	1.000	0.857	1.000	NA, NA
	2	1.000	1.000	1.000	NA, NA
	3	1.000	1.000	1.000	NA, NA
<5 cm	1	0.538	0.615	0.538	> 0.99, > 0.99
	2	0.385	0.462	0.462	> 0.99, > 0.99
	3	0.308	0.231	0.231	> 0.99, > 0.99
Diameter					
> 7 mm	1	0.818	0.727	0.727	> 0.99, > 0.99
	2	0.818	0.818	0.818	NA, NA
	3	0.727	0.727	0.727	NA, NA
< 7 mm	1	0.556	0.667	0.667	> 0.99, > 0.99
	2	0.333	0.444	0.444	> 0.99, > 0.99
	3	0.333	0.222	0.222	> 0.99, > 0.99
Location					
Femoral	1	1.000	0.750	1.000	NA, NA
	2	1.000	1.000	1.000	NA, NA
	3	1.000	1.000	1.000	NA, NA
Peripheral	1	0.625	0.688	0.625	> 0.99, > 0.99
	2	0.500	0.563	0.563	> 0.99, > 0.99
	3	0.438	0.375	0.375	> 0.99, > 0.99
Occlusion					
Complete	1	0.750	0.625	0.750	> 0.99, NA
	2	0.750	0.750	0.750	NA, NA
	3	0.750	0.750	0.750	NA, NA
Partial	1	0.667	0.750	0.667	> 0.99, > 0.99
	2	0.500	0.583	0.583	> 0.99, > 0.99
	3	0.417	0.333	0.333	> 0.99, > 0.99

Comparisons were conducted using McNemar’s test

DLR deep learning reconstruction; FBP, filtered back projection; IR, iterative reconstruction

**Table 6:** Results of qualitative image analysis

	Reader	DLR	Hybrid IR	FBP	*P* value (vs. Hybrid IR, vs. FBP)
DVT lesion depiction	1	9/15/6/0	8/8/13/1	0/6/20/4	0.020 *, < 0.001 *
	2	14/12/3/1	5/13/10/2	4/8/14/4	< 0.001 *, < 0.001 *
	3	13/7/5/5	11/11/3/5	12/8/6/4	0.877, > 0.99
Structure depiction (vein)	1	33/10/7/1	6/24/19/2	0/19/25/7	< 0.001 *, < 0.001 *
	2	30/20/1/0	14/27/9/1	13/24/13/1	< 0.001 *, < 0.001 *
	3	46/4/1/0	9/39/3/0	9/33/8/1	< 0.001 *, < 0.001 *
Structure depiction (muscle)	1	40/9/2/0	2/37/11/1	0/18/32/1	< 0.001 *, < 0.001 *
	2	41/10/0/0	20/30/0/1	14/34/2/1	< 0.001 *, < 0.001 *
	3	50/1/0/0	10/39/2/0	12/35/3/1	< 0.001 *, < 0.001 *
Subjective image noise	1	34/16/0/1	0/20/30/1	0/2/32/17	< 0.001 *, < 0.001 *
	2	40/11/0/0	7/38/6/0	1/26/23/1	< 0.001 *, < 0.001 *
	3	27/20/4/0	1/24/25/1	0/9/35/7	< 0.001 *, < 0.001 *
Artifacts	1	10/33/8	1/37/13	2/28/21	0.005 *, < 0.001 *
	2	40/7/4	38/8/5	34/10/7	0.407, 0.025
	3	40/7/4	9/36/6	8/31/12	< 0.001 *, < 0.001 *
Overall image quality	1	16/20/13/1/1	0/2/31/17/1	0/0/11/32/8	< 0.001 *, < 0.001 *
	2	25/21/4/1/0	8/18/18/6/1	3/15/21/11/1	< 0.001 *, < 0.001 *
	3	26/22/2/1/0	0/11/36/3/1	1/7/29/13/1	< 0.001 *, < 0.001 *

The numbers of patients for each score (5/4/3/2/1, 4/3/2/1, or 3/2/1) are shown

DVT, deep vein thrombosis; DLR, deep learning reconstruction; FBP, filtered back projection; IR, iterative reconstruction

*, statistically significant difference

## Discussion

Our study aimed to compare DLR with Hybrid IR and FBP in terms of lesion detection and image quality for contrast-enhanced CT venography in patients undergoing evaluation for DVT. Our findings suggest that DLR significantly improves image quality and, for some readers, also enhances lesion visibility compared to Hybrid IR and FBP.

Our results demonstrated that DLR reduced image noise and enhanced the CNR when compared to both Hybrid IR and FBP. These results are consistent with prior studies demonstrating that DLR can effectively reduce image noise and enhance image quality in CT imaging [21–23]. While our study found no significant differences in AUC or sensitivity for lesion detection among DLR, Hybrid IR, and FBP, DLR demonstrated improved image quality and reduced image noise. These improvements may help reduce the diagnostic burden on radiologists. This finding aligns with some studies suggesting no significant improvement in lesion detection with the use of DLR [21, 22]. Another study on hepatocellular carcinoma demonstrated significant improvement [23]. The reason for this discrepancy may be that the liver can experience morphological irregularities and attenuation inconsistencies due to diseases such as cirrhosis, whereas the lower limb veins tend to maintain a relatively linear morphology and are often more uniform, making them easier to observe. Future research is needed to investigate the factors that influence detectability. Another possible explanation for the limited improvement in sensitivity and AUC for DVT, despite the enhanced image quality with DLR, could be a potential ceiling effect—where the already reasonably high conspicuity of DVT may limit the extent to which further gains in diagnostic performance can be observed.

The relatively low sensitivity scores and AUC for the two resident readers (Reader 2 and 3) compared to the radiologist (Reader 1) underscore the influence of reader experience on lesion detection performance. The moderate inter-reader agreement (Fleiss’ kappa: 0.417) further highlights this variability, pointing to the potential for diagnostic inconsistency, especially among less experienced readers.

In general, a reduced tube current results in a lower signal-to-noise ratio. Although our study did not specifically evaluate reductions in tube current, the improved image quality achieved with DLR suggests the potential for lowering the tube current. The same may also apply to reductions in contrast medium. Future study needs to focus on this topic.

One limitation of our study is its retrospective design, which was conducted at a single institution with a relatively small sample size. Additionally, we focused on DVT lesions in the lower limbs, and our results may not be generalizable to other venous or arterial pathologies. Future studies with larger, multi-center cohorts and diverse lesion types are needed to better assess the generalizability of our findings. Another limitation of our study is the absence of longitudinal follow-up. The clinical utility of improved image quality and depiction of DVT lesions—specifically, whether it can help prevent the development of PE—remains to be investigated. The potential learning curve bias among readers who were not initially familiar with DLR images could also be limitation. With repeated reading experiments, some readers may become more accustomed to DLR images, while others may not, introducing variability in their interpretations. This risk may persist even when intervals are placed between each experimental session. Furthermore, as this study used a DLR algorithm from a single vendor, the results may not apply to DLR algorithms from other manufacturers. However, noise reduction on CT images of various body parts has also been reported for other vendors, such as TrueFidelity (GE Healthcare, Waukesha, USA) and Precise Image (Philips Healthcare, Cleveland, USA) [24, 25]. This study could potentially be applied to their technologies as well.

Our results suggest directions for future research. One promising approach is the application of radiomics to DLR-enhanced images to improve thrombus characterization. This analysis may facilitate the identification of imaging biomarkers that could predict treatment outcomes. Another area of exploration could be the use of DLR in non-contrast venography or ultra-low-dose CT protocols. Although such CT images tend to be of low quality, DLR’s denoising capabilities can improve thrombus visualization. Lastly, the integration of DLR into automated algorithms for real-time DVT detection could be a transformative step forward. Machine learning algorithms, when coupled with DLR-enhanced images, could improve triage and decision-making in clinical settings.

In conclusion, DLR substantially enhances the quality of images by reducing noise and accentuating the visualization of both vascular structures and normal anatomy. While DLR did not show significant improvements in lesion detection sensitivity within our cohort, some readers perceived it as offering better visual clarity of DVT, and all readers noted a degree of noise reduction. These findings indicate that DLR can be a valuable instrument in improving the diagnostic quality of contrast-enhanced CT venography for DVT, potentially facilitating clinical assessments and management.

## Electronic supplementary material

Below is the link to the electronic supplementary material.

Supplementary Material 1

## Data Availability

My manuscript has no associated data.
