# Supplementary material for: Deep learning reconstruction enhances image quality in contrast-enhanced CT venography for deep vein thrombosis
Source: Emerg Radiol. 2025 Jul 18;32(5):699–708. doi: 10.1007/s10140-025-02366-x (PMC12496264; doi:10.1007/s10140-025-02366-x)
Supplement: Supplementary file 1 — Supplementary Material 1 [file 10140_2025_2366_MOESM1_ESM.pptx]

## Slide 1
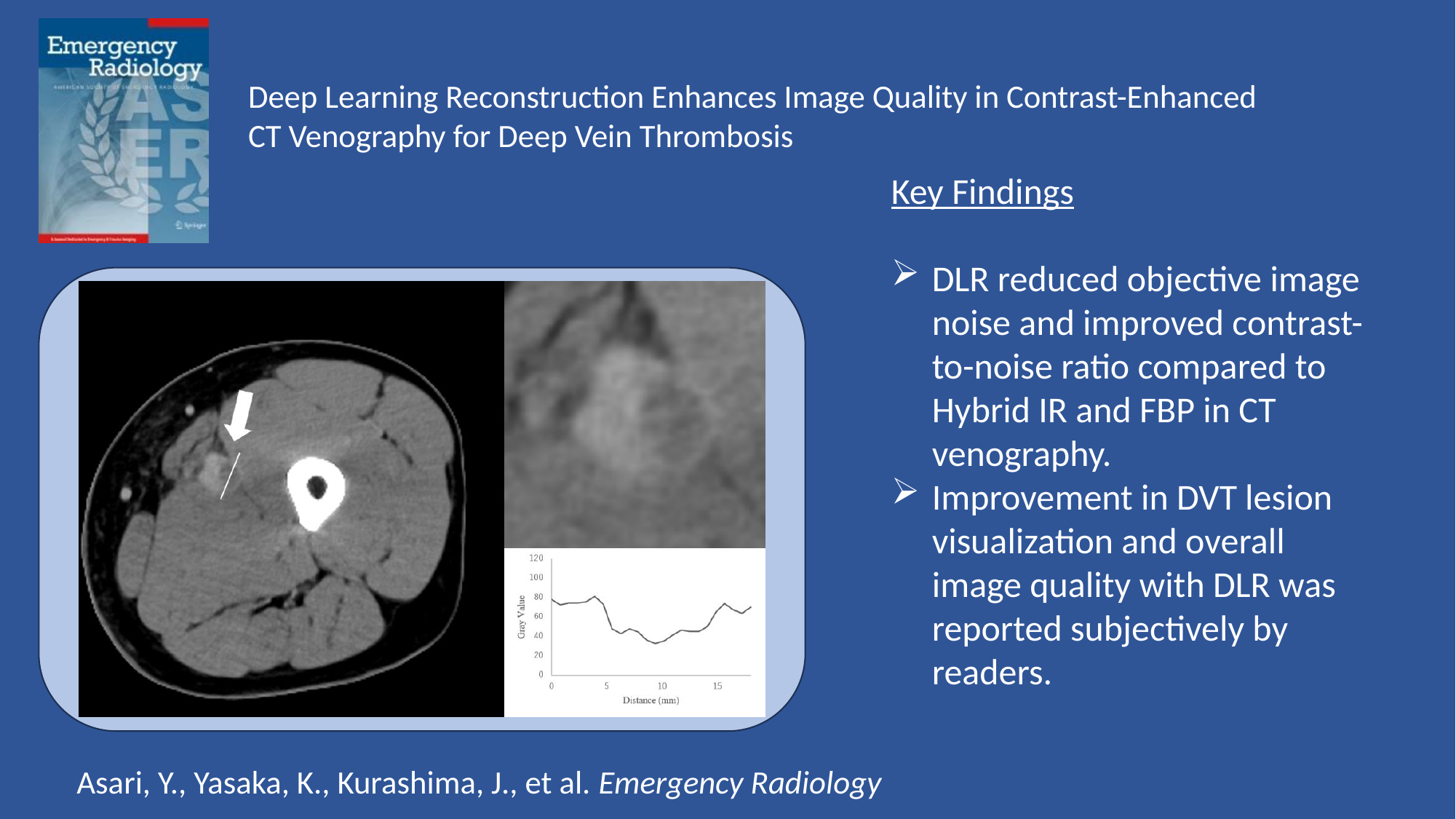

Deep Learning Reconstruction Enhances Image Quality in Contrast-Enhanced
CT Venography for Deep Vein Thrombosis
Key Findings
DLR reduced objective image noise and improved contrast-to-noise ratio compared to Hybrid IR and FBP in CT venography.
Improvement in DVT lesion visualization and overall image quality with DLR was reported subjectively by readers.
Asari, Y., Yasaka, K., Kurashima, J., et al. Emergency Radiology
